# Alcohol and electric scooter injuries in an emergency department: a prospective observational study

**DOI:** 10.1186/s13049-025-01427-x

**Published:** 2025-07-01

**Authors:** Jenny Liu, Alexandra Rajevic, Philip von Arbin, Kristian Ängeby

**Affiliations:** Department of Clinical Science and Education Södersjukhuset, Karolinska Institutet, Stockholm, 17177 Sweden

**Keywords:** Alcoholic beverages, Electric scooter, Injuries, Traffic accident, Emergency department

## Abstract

**Background:**

Variable prevalences of alcohol use have been reported among injured electric scooter (e-scooter) riders in emergency departments (ED) worldwide. The studies indicate a relationship between alcohol and injuries, especially at nighttime. A better understanding would guide the prevention of these injuries. The study objectives were to investigate the relationship between alcohol and injury patterns as well as the impact of restriction policies on ED presentations.

**Methods:**

A prospective observational study at an urban level 2 trauma centre ED, where all patients presenting with e-scooter injuries from April to September 2022 were eligible and asked for consent to participate and to take a breathalyser test. Pearson´s chi-squared test and Mann-Whitney U test were used for comparison between participants with/without alcohol involvement. In addition, the proportion of e-scooter injuries before and after restriction policies were introduced in 2022 was analysed together with city data on the number of rental e-scooters and trips 2021–2023.

**Results:**

One patient declined, and 133 injured riders consented. They were younger (mean age 34.1 years) and more often male (66.2%) compared to other ED patients. Alcohol was involved for 57/133 (42.9%) participants and data was missing for 25 (18.8%) participants. Alcohol use was more prevalent among participants arriving at night (22:00–05:59), 43/68 (63%; CI: 51–75) compared to 14/65 (22%; CI: 11–32) participants arriving between 06:00–21:59. Face and head injuries were more frequent with alcohol use, 44/57 (77%; CI: 66–88) compared to 29/51 (57%; CI: 43–71) without. However, the median injury severity score was lower with alcohol use. After the introduction of a city limit on the number of rental e-scooters, the number of trips in the city decreased by 35% and a 39% reduction of e-scooter injuries was found in the study ED.

**Conclusions:**

Alcohol use was more prevalent among e-scooter riders presenting nighttime to the ED. Face and head injuries were more frequent with alcohol use. E-scooter city trips and injuries in the ED were reduced when the number of rental e-scooters had been limited. Future studies should evaluate interventions targeting intoxicated nighttime riding and helmet use.

## Introduction

Stand-up electric scooters (e-scooters) have rapidly gained popularity worldwide since the introduction of short-term rentals accessible on digital platforms in 2017 [1]. The surge in e-scooter use has been accompanied by an increase in reported injuries. A 2022 systematic review of 34 studies indicated that e-scooter incidents often result in head injuries and fractures, predominantly in the limbs, causing substantial direct medical costs [2]. These findings have been confirmed by recent studies worldwide [3, 4], including in the Nordic capitals [5–10]. Other consistent findings are that injured e-scooter riders are younger than the average patient in the emergency department (ED), predominantly male, and seldom wear a helmet [2].

However, there is a large variation in the literature on the prevalence of alcohol intoxication in e-scooter incidents, ranging from 3% [11] to 45% [6] in ED cohorts and from 51 to 74% in cohorts from maxillofacial or neurosurgery trauma centres [12–14]. In most studies, data have been collected retrospectively from medical records or registries, which may underestimate alcohol involvement. Only a few studies have prospectively collected alcohol data and usually in terms of patient-reported alcohol consumption prior to the injury [7, 9, 15, 16].

Although e-scooter injuries in Sweden have been previously reported in three studies, there are two reasons to conduct a new study. These studies were based on retrospective data from claims made to an insurance company [8], the Swedish Traffic Accidents Data Acquisition (STRADA) registry [5, 8], and the Swedish Fracture Register [18]. These were registries with limited coverage requiring manual data entry. Moreover, in the STRADA registry used by two of the studies [5, 8] only a subset of patients who filed an incident report were entered before the enactment of a new law in July 2021 [17], while the third study only investigated orthopaedic fractures [18].

The increase in e-scooter incidents has led to the introduction of various regulations and restrictions. Only a few studies have evaluated the impact of such interventions, such as mandatory helmet laws [19], a reduced number of vehicles [20], or a combination of speed limits and rental prohibitions [21–23]. The city of Paris voted to ban all rentals in 2023, with Melbourne following suit in 2024 [24]. In Sweden, the City of Stockholm imposed a limit on the number of rental e-scooters in January 2022 [25], which was followed by a national ban on e-scooter riding and parking on pavements and walkways in September 2022 [26]. Evaluating these interventions is another reason to conduct a new Swedish study.

## Methods

### Aim

The aim of this study is twofold. First, to prospectively investigate e-scooter riders presenting to an ED in Stockholm regarding their alcohol use and injury pattern. Second, to evaluate the impact of the imposed restriction policies on ED visits.

### Study design and setting

A prospective observational study was conducted from 1 April to 30 September 2022 at the adult ED of Södersjukhuset, a 600-bed general hospital and level 2 trauma centre located in central Stockholm, Sweden. The ED receives adult trauma patients from the age of 15 years. The trauma care in Stockholm County is organized in one level 1 trauma centre and six level 2 centres operating 24/7, while urgent care centres receive patients with minor injuries daily from 08:00 to 22:00. During the study period, 318 e-scooter riders injured in Stockholm County were entered into the STRADA registry. Of these, 38% were treated at the study hospital, 23% at the other level 2 centres, and 8% at the level 1 trauma centre. The remaining 31% were treated at multiple urgent care centres. The Injury Severity Score (ISS) [27] was 1–8 for 92% of the injured e-scooter riders and 9–15 for 5%. The remaining 3% of the participants with ISS > 15 were all treated at the level 1 trauma centre.

### Participants

During the study period, all patients presenting to the ED with e-scooter injuries were eligible. Eligible patients were handed an information sheet with an inquiry for consent to participate. The ED triage staff was instructed to ask these patients to take an alcohol breathalyser test, unless a physician had already ordered a serum ethanol. Considering that breath alcohol is detectable for 12–24 h [28], only patients presenting to the ED within 24 h of the incident were asked to take the test.

### Data collection

To prospectively investigate e-scooter riders presenting to the ED, the background variables of all ED patients, including chief complaint, trauma/non-trauma, age, gender, and arrival time, were collected from the ED electronic tracking system *TakeCare Akutliggare* (CampuGroup Medical Sweden AB) via the *Qlikview*^®^ business intelligence tool (Qlik, Pennsylvania, USA). The breathalyser used was the Lion Alcolmeter^®^ 700 (Lion Laboratories Limited, UK) with a measurement range of 0.02–2.00 mg/L breath alcohol content. The instrument returned the test result in mg/g (‰) blood alcohol concentration (BAC). The BAC variable was collected from the participants’ electronic medical records along with the sustained injury type and body region. The ISS was then calculated according to Abbreviated Injury Scale (AIS) version 2005 updated 2008.

To ensure consistent data entry, we applied the principle that a diagnosed fracture also implies a contusion of the affected area. In such cases, only the fracture was entered. This was also applied to trauma to the head, so that contusion was entered if a participant sustained cerebral concussion with no other brain injury. The AIS was 2 for patients with loss of consciousness and 1 without in the AIS version used. Additional variables collected from the medical records were helmet use, time of the incident, procedures, and follow-up.

The City of Stockholm imposed a limit of 12,000 rental e-scooters in January 2022 after the number exceeded 23,000 in September 2021 [25]. In September 2022, the Swedish Transport Agency introduced a national ban on e-scooter riding and parking on pavements and walkways [26]. To evaluate the impact of these restriction policies, the study used the number of e-scooter injuries in the ED from April to September during the years 2021 to 2023 together with the number of rental e-scooters and trips in the city during the corresponding periods. These data were collected from the ED tracking system via the Qlikview^®^ tool and from the rental e-scooter data published weekly by the City of Stockholm [25]. We minimized the effects of different weather conditions by basing the comparison on a time period when road surfaces were free from snow and the air temperature exceeded zero degrees Celsius.

### Data analysis

All collected data were anonymised and transferred to IBM SPSS Statistics version 29.0.1.0 for analysis. Participant characteristics were reported using descriptive statistics. Mean values of normally distributed variables, such as age and BAC, were compared using Student’s t-test, while the Mann-Whitney U test and bootstrapping with 1,000 samples was applied for ISS. Proportions were compared using Pearson´s chi-squared test. Breathalyser results above BAC 0.00‰ were regarded as positive and 0.00‰ as negative, while serum test results in mmol/l were multiplied by 0.0363 for conversion to ‰ BAC [29]. We compared alcohol use depending on the variables age, sex, nighttime and weekend arrivals, AIS, and ISS. A 95% confidence interval was reported where appropriate and the two-tailed statistical significance level was set at 0.05.

## Results

**Fig. 1:**
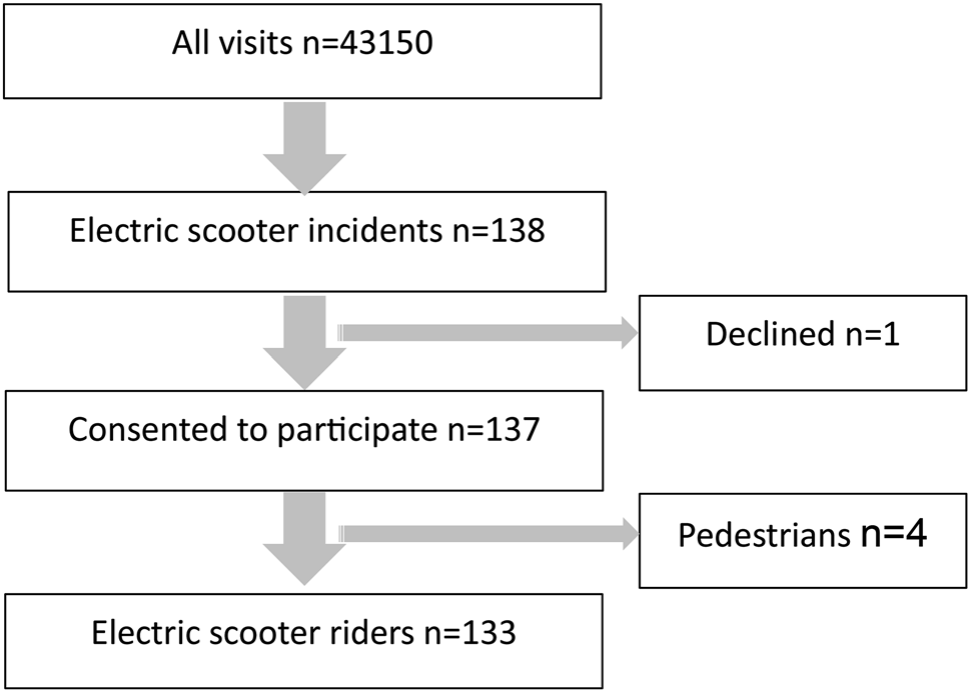
Flow chart of patient inclusion

There was a total of 43,150 ED visits during the study period from 1 April to 30 September 2022. Among all ED visits, 138 (0.3%) were caused by e-scooter incidents. Of the eligible patients, 133 e-scooter riders were included in the study, since one did not give consent and four were pedestrians (Fig. 1).

### Participant characteristics

Among the included e-scooter riders, the mean age was 34.1 years (CI: 31.9–36.4) and 66.2% (88/133; CI: 58.1–74.3%) were men. The participants were younger and more often male compared to other trauma patients (*n* = 5,790) and even more so when compared to non-trauma patients (*n* = 37,227) in the ED. Moreover, a larger proportion of the participants presented to the ED during nighttime from 22:00 to 05:59, 51.1% (68/133; CI: 42.5–59.5%) compared to other trauma patients and non-trauma patients. However, the proportion of patients presenting during weekends from Saturday to Sunday did not differ between the participants and the other two patient categories (Table 1). Eleven (8.3%) participants reported helmet use and 74 (55.6%) reported no helmet, while the data was missing for the remaining 48 (36.1%) participants.

**Table 1:** Characteristics of study participants versus other trauma presentations and non-trauma presentations during the study period

	E-scooter riders	Other trauma presentations	Non-trauma presentations
**Number**	133	5790	37,227
**Age**			
Mean (SD), years	34.1 (13.2)	57.4 (24.2)	58.4 (21.3)
95% CI, years	31.9–36.4	56.8–58.1	58.1–58.6
P-value		< 0.001	< 0.001
**Sex**			
Female, n (%)	45 (33.8)	2775 (47.9)	18,518 (49.7)
Male, n (%)	88 (66.2)	3015 (52.1)	18,709 (50.3)
95% CI, male (%)	58.1–74.3	50.8–53.4	49.8–50.8
P-value		0.001	< 0.001
**Arrival time**			
06:00–21:59, n (%)	65 (48.9)	4106 (70.9)	29,909 (80.3)
22:00–05:59, n (%)	68 (51.1)	1684 (29.1)	7318 (19.7)
95% CI, 22:00–05:59 (%)	42.5–59.5	27.8–30.2	19.6–20.4
P-value		< 0.001	< 0.001
**Arrival day**			
Monday to Friday, n (%)	93 (69.9)	4017 (69.4)	28,300 (76.0)
Saturday to Sunday, n (%)	40 (30.1)	1773 (30.6)	8927 (24.0)
95% CI, Saturday to Sunday (%)	22.2–37.8	29.8–32.2	23.5–24.5
P-value		0.892	0.100

### Alcohol involvement

Of 133 participants, 126 presented to the ED within 24 h of the incident and 71% (89/126) took an alcohol test in which 51% (45/89) tested positive. In addition, of those testing negative, three participants reported that they had used alcohol. Also, alcohol use was reported by eight participants who did not take an alcohol test and one participant who presented more than 24 h after the incident. In total, 42.8% (57/133) participants were known to have used alcohol, while 38% (51/133) tested negative or denied alcohol use. The information was missing for the remaining 18.8% (25/133). There was no significant difference in age, sex, arrival time or day between the participants whose data were missing and the other 108 participants. These characteristics did not differ significantly between the participants who did not take an alcohol test (29.4%, 37/126) and those who did (70.6%; 89/126) (Appendix 1).

**Fig. 2:**
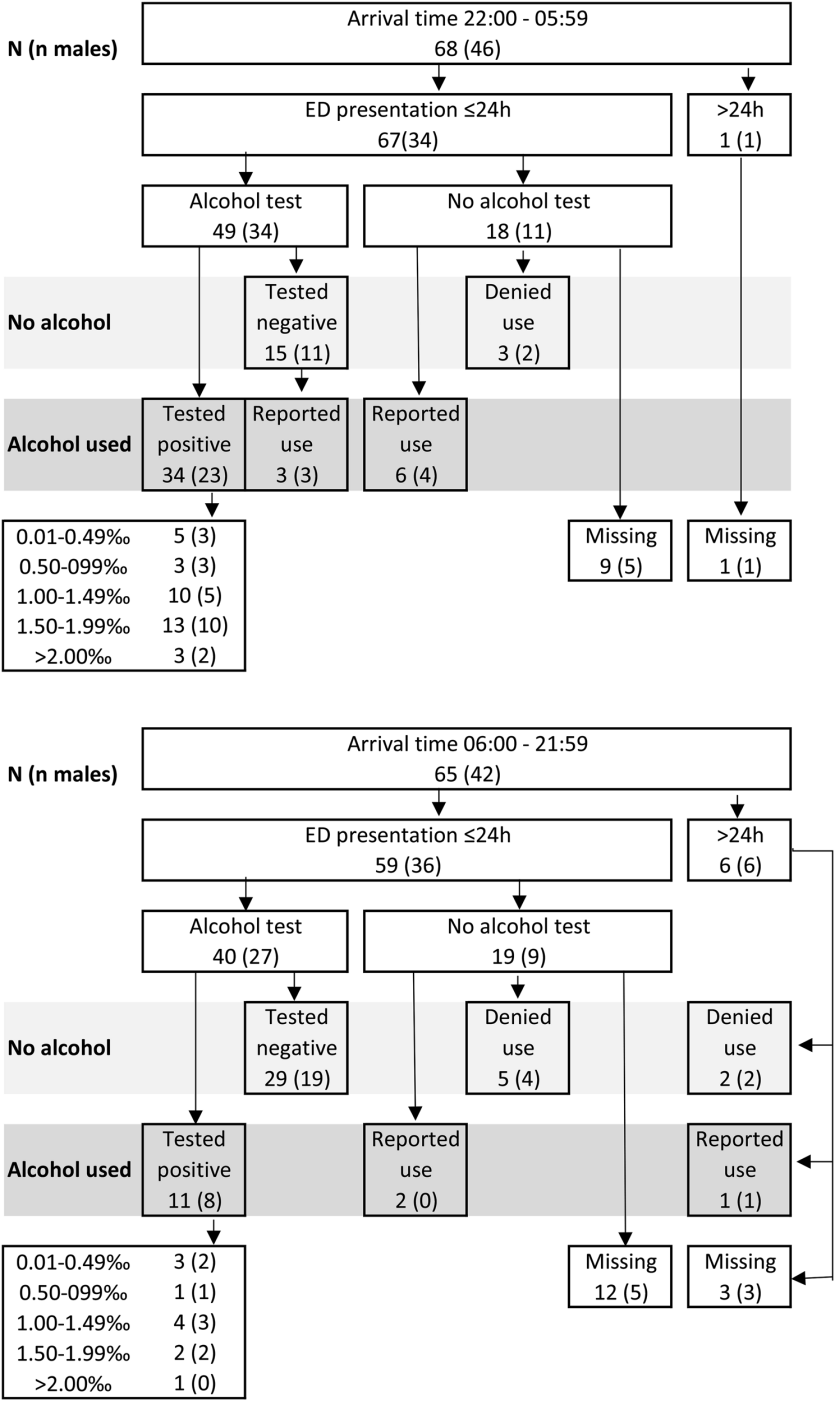
Alcohol involvement and arrival time. Number of participants presenting nighttime 22:00–05:59 and the remaining hours, respectively. The number of male participants is presented in parenthesis

**Fig. 3:**
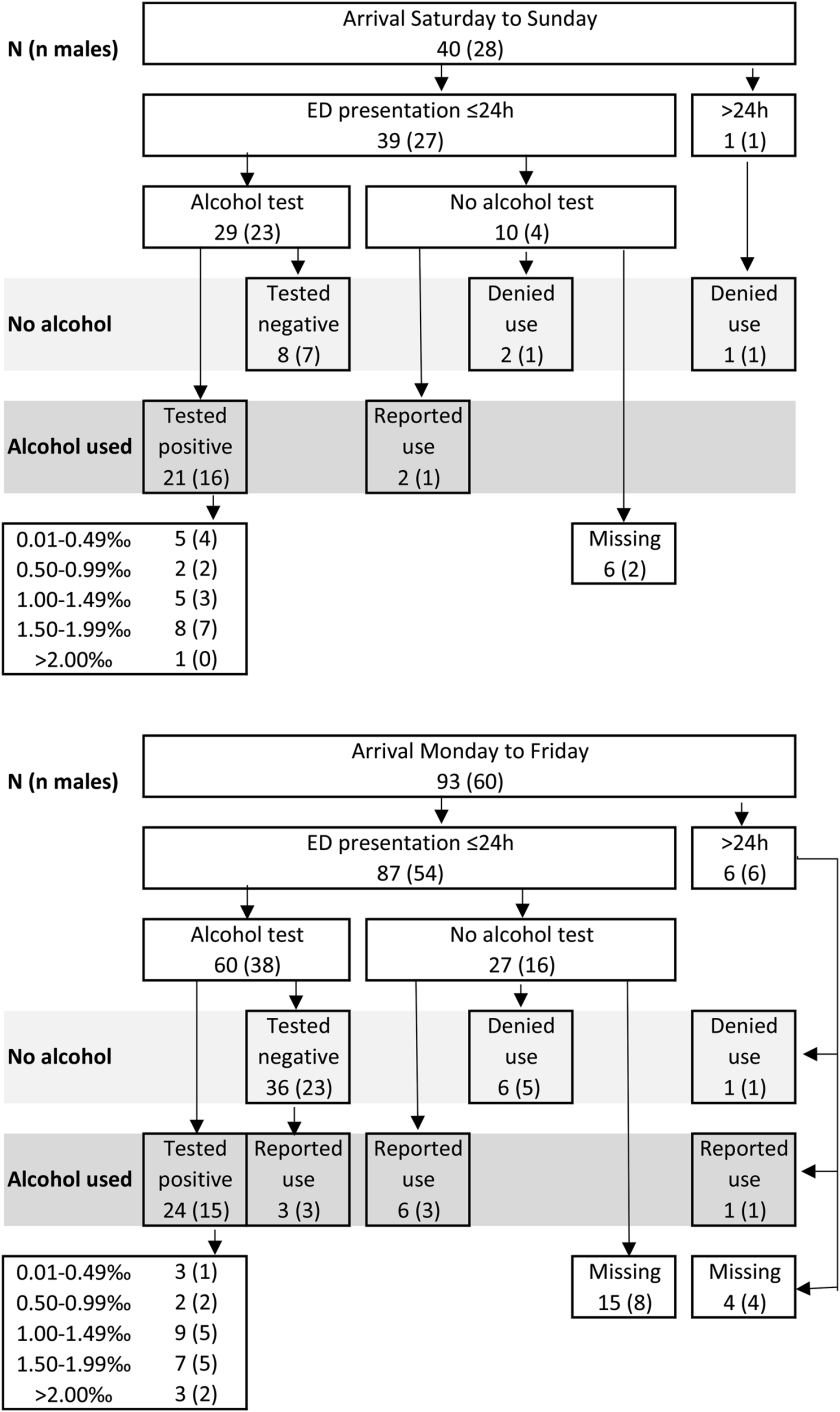
Alcohol involvement and arrival day. Number of participants presenting on weekends and weekdays, respectively. The number of male participants is presented in parenthesis

Alcohol was more often involved among the participants who arrived to the ED at nighttime between 22:00 and 05:59, 63% (43/68; CI: 51–75%) compared to 22% (14/65; CI: 11–32%) among those arriving between 06:00 and 21:59, hereafter referred to as daytime (*p* < 0.01). Of those arriving at nighttime, 22% (15/68) tested negative or denied alcohol use, compared to 55% (36/65) of those arriving during daytime. This information was missing for 15% (10/68) of the nighttime arrivals and for 23% (15/65) of the daytime arrivals (Fig. 2). Additionally, alcohol was more often involved among the participants who arrived on weekends, 58% (23/40; CI: 41–74) compared to 37% (34/93; CI: 27–47) among weekday arrivals (*p* = 0.025). This information was missing for 15% (6/40) of the participants who arrived on weekends and 20% (19/93) among weekday arrivals (Fig. 3).

However, when comparing only participants who tested positive for alcohol, no significant difference was found in the mean BAC depending on arrival time or day. The mean BAC was 1.33‰ (CI: 1.14–1.51‰) among 34 nighttime arrivals and 1.11‰ (CI: 0.75–1.48‰) among eleven daytime arrivals (*p* = 0.546). The mean BAC was 1.18‰ (CI: 0.91–1.45‰) among 21 weekend arrivals and 1.35‰ (CI: 1.12–1.59‰) among 24 weekday arrivals (*p* = 0.316).

Likewise, there was no difference in alcohol involvement regarding age or sex. The mean age was 33.8 years (CI: 30.7–36.9) among the 57 participants who had used alcohol and 34.4 years (CI: 31.2–37.6) among the 51 who had not (*p* = 0.800). Among the male participants, 44% (39/88; CI: 34–55%) had used alcohol compared to 40% (18/45; CI: 25–55%) among the female participants (*p* = 0.634). Among the participants testing positive for alcohol, the mean BAC was 1.27‰ (CI: 1.06–1.48‰) for 31 males and 1.29‰ (CI: 0.94–1.64‰) for 14 females (*p* = 0.859).

### Injury pattern

A total of 238 injuries were recorded among 133 participants with 64.7% (86/133) sustaining more than one injury. The body regions most frequently injured were the face (32.8%; 78/238) and upper limb (24.8%; 59/238), followed by the head (23.1%; 55/238), lower limb (13.4%; 32/238;), and torso (5.9%; 14/238). The most frequent injury type was contusion (45.8%; 109/238), followed by fracture (29.0%; 69/238) and laceration (15.1%; 36/238). The fractures were most frequent in the upper limb (45%; 31/69), face (26%; 18/69), and lower limb (19%; 13/69) (Table 2). The maximum AIS was 1 for 54.1% (72/133) of the participants, 2 for 39.1% (52/133), and 3 for 6.8% (9/133). Consequently, the ISS was 1–8 for 92.5% (123/133) of the participants and 9–15 for 7.5% (10/133). No participant had maximum AIS over 3 or ISS over 15.

**Table 2:** Overall injury pattern - Injury type and body region among all 133 participants

	Face	Head	Upper limb	Lower limb	Torso	Sum *n* (%)
Contusion	31	38	16	14	10	109 (45.8)
Fracture	18	4	31	13	3	69 (29.0)
Laceration, sutured	19	6	2	1		28 (11.8)
Laceration, not sutured	3	1	4			8 (3.4)
Dental injury	7					7 (2.9)
Intracranial hemorrhage		6				6 (2.5)
Joint dislocation			6			6 (2.5)
Distorsion				4		4 (1.7)
Pneumothorax					1	1 (0.4)
**Sum n (%)**	**78 (32.8)**	**55 (23.1)**	**59 (24.8)**	**32 (13.4)**	**14 (5.9)**	**238 (100)**

A smaller proportion of limb injuries was found among the participants who had used alcohol, 40% (23/57; CI: 27–53%) compared to 67% (34/51; CI: 53–80%) among those who had not (*p* = 0.006). Conversely, a larger proportion of face and head injuries was found among the participants who had used alcohol, 77% (44/57; CI: 66–88%) compared to 57% (29/51; CI: 43–71) among those who had not (*p* = 0.024) (Table 3).

There was a larger proportion of maximum AIS 1 among participants who had used alcohol compared to those who had not (*p* = 0.028). Consequently, the median ISS was 2 (CI: 1–2) for those who had used alcohol, compared to 4 (CI: 2–4) for those who had not (*p* = 0.023). On the other hand, no significant difference was found between the 25 participants whose alcohol data were missing and the other 108 participants in terms of the maximum AIS distribution (*p* = 0.086) or the median ISS (4; CI: 2–4 and 2; CI: 2–4, respectively) (*p* = 0.409) (Appendix 2).

**Table 3:** Injury pattern among participants with and without alcohol involvement

Alcohol involved (*n* = 57)	Face	Head	Upper limb	Lower limb	Torso	Sum *n* (%)
Contusion	19	17	5	5	3	49 (47)
Fracture	11	1	8		2	22 (21)
Laceration	14	3	3			20 (19)
Intracranial hemorrhage		3				3 (3)
Joint dislocation				2		2 (2)
Distorsion			4			4 (4)
Dental injury	4					4 (4)
Pneumothorax					1	1 (1)
**Sum n (%)**	**48 (46)**	**24 (23)**	**20 (19)**	**7 (7)**	**6 (6)**	**105 (100)**
**No alcohol use **(***n = 51)***	**Face**	**Head**	**Upper limb**	**Lower limb**	**Torso**	**Sum n (%)**
Contusion	6	14	8	5	5	39 (44)
Fracture	7	3	16	6	1	33 (38)
Laceration	3	3	2	1		9 (10)
Intracranial hemorrhage		3				3 (3)
Joint dislocation			2			2 (2)
Distorsion				1		1 (1)
Dental injury	1					1 (1)
Pneumothorax						0
**Sum n (%)**	**17 (19)**	**23 (26)**	**28 (32)**	**14 (16)**	**6 (7)**	**88 (100)**

The hospitalization rate did not differ significantly between the participants who had used alcohol (7%; 4/57; CI: 0–14%), those who had not (18%; 9/51; CI: 7–28%), and those whose alcohol data was missing (20%; 5/25; CI: 3–37%). In total, 13.5% (18/133) were hospitalized, but no participants required intensive care. Of those hospitalized, 94% (17/18) sustained fractures and 44% (8/18) required in-patient orthopedic surgery. Out of the six participants with intracranial hemorrhage and one with pneumothorax, none required surgical intervention. Among the hospitalized patients, the median ISS was 8 (CI: 4–9) compared to ISS 2 (CI: 2–3) for the non-hospitalized participants (*p* < 0.001). Of the non-hospitalized participants, 37% (42/115) sustained fractures, 28.7% (33/115) were followed up by various surgery specialties, and 8% (9/115) required surgical intervention. In total, 12.8% (17/133) required surgical interventions after the ED treatment, which often included wound suturing, reduction and cast immobilization of dislocations and fractures.

### Impact of restriction policies on ED presentations

The City of Stockholm introduced a limit of the number of rental e-scooters in January 2022, followed by a national ban on e-scooter riding and parking on walkways in September 2022. Prior to these restrictions, 0.51% (211/41,675; CI 0.44–0.57%) of the ED presentations from 1 April to 30 September 2021 were registered as e-scooter injuries. There was a significant reduction to 0.32% (138/43,164; CI 0.26–0.36%) e-scooter injuries during the corresponding period in 2022 (*p* < 0.001). In 2023, the decrease to 0.28% (121/42,946; CI 0.23–0.33%) e-scooter injuries during the corresponding period was not statistically significant (*p* = 0.475). According to weekly data published by the City of Stockholm, the average daily number of rental e-scooters during the period from April to September decreased from 19,100 in 2021 to 9,200 in 2022 and increased to 9,600 in 2023. During the corresponding periods from April to September, the total number of trips on rental e-scooters in Stockholm decreased from 8.9 million in 2021 to 5.8 million in 2022, and to 4.0 million in 2023 [25].

## Discussions

We investigated alcohol involvement among injured e-scooter riders presenting to the ED and found that four out of ten participants had used alcohol. Among participants presenting at nighttime or on weekends, six out of ten had used alcohol. Eight out of ten participants who had used alcohol sustained face and head injuries. We also evaluated the impact of restriction policies and found a reduction of e-scooter injuries in the ED by one third and a corresponding decrease in the total number of rental e-scooter trips in the city after the introduction of a limit on the number of rental e-scooters.

### Alcohol involvement

By systematically screening for alcohol, we found a prevalence rate higher than the 3–20% found in previous ED studies from non-Nordic countries [11, 15, 30, 31] and the 28% found in a Stockholm registry study [5]. However, it is in the same range as that of 37–45% found in other Nordic EDs [6, 7, 9, 32]. Varying designs may have contributed to the different alcohol prevalence rates in previous studies, for example, Genc Yavuz et al. retrospectively collected documented blood alcohol levels [11]. The prevalence rates also varied among the few prospective studies, from a self-reported alcohol prevalence of 40% in Reykjavik, Iceland [9], to 20% in Berlin, Germany [15], and to 15% when participants were only tested for blood alcohol on legal grounds [16]. Naturally, cultural factors and alcohol policies also contribute to varying prevalence rates. In line with previous studies [6, 7], alcohol use was more prevalent among e-scooter riders presenting to the ED at nighttime or on weekends.

### Injury pattern

A proportion of 49–58% head and face injuries has also been reported in several studies [6, 31, 34], while other studies have found a smaller proportion of 32–41% [7, 11, 15]. The larger proportion of face and head injuries found among participants who had used alcohol agrees with the 51–74% alcohol prevalence rates reported from neurosurgery and maxillofascial settings [12–14]. Mortal traumatic brain injury has been reported in a neurosurgery cohort [12], but has otherwise been rare among ED cohorts. Across studies, the helmet usage rate was as low as 0–6% among adults [2, 35]. Benhamed et al. found a smaller proportion of AIS ≥ 1 face/head injuries among e-scooter riders who had worn a helmet compared to those who had not [35]. Despite a mandatory e-scooter helmet law and a 33% helmet usage rate, the proportion of head and face injuries was 50% among injured e-scooter riders in Melbourne, Australia [4]. The helmet use increased from 2 to 12%, but only during morning and afternoon hours, after the introduction of a mandatory helmet law in Denmark [36]. Future studies should be designed to screen helmet use to allow analysis of helmet use and injury pattern.

The injury pattern agrees in several other aspects with previous studies, for example, the upper limbs were more affected than the lower limbs [2]. Similar rate of fractures [6, 15, 37] and surgical interventions [6, 9, 34] have also been found. The small proportion of dental injuries is supported by Stigson et al., who found that such injuries were more often reported to the insurance company than to the STRADA registry, indicating that e-scooter riders with isolated dental injuries did not present to the ED [8]. As in the few studies that have used AIS to describe the injuries [6, 7], over 90% of the e-scooter riders sustained only minor injuries with ISS 1–8 and AIS 1–2. To the best of our knowledge, an analysis of AIS/ISS and alcohol involvement has not been reported previously. The lower median ISS we found among the participants who had used alcohol reflected the fact that cerebral concussion without loss of consciousness was coded as AIS 1 in the 2008 version, while the more prevalent limb fractures among participants who had not used alcohol were mostly coded as AIS 2.

### Impact of restriction policies on ED presentations

Markowitz et al. also found a decrease in e-scooter injuries from 2.8 to 1.8% at a level 1 trauma centres when the City of Miami revoked the permit for several e-scooter companies [20]. However, other factors may have contributed to the reduction of e-scooter injuries and city trips in this study. A direct causality cannot be claimed, though we minimized the effects of different weather conditions by comparing a period when road surfaces were free from snow and ice [38]. Fewer e-scooter injuries were also seen in Helsinki EDs when a lower speed limit was combined with prohibited use on Friday and Saturday nights [23]. On the other hand, some studies did not find a decrease of e-scooter injuries, for example, with a reduced speed limit only during nighttime in Tampere, Finland [22], or a nighttime rental ban in Atlanta, USA [21]. Further research is needed on the impact of various interventions to minimize e-scooter injuries, such as stricter laws or incentives to increase sobriety and helmet use. In an interview study, injured e-scooter riders also suggested pre-ride briefings and designated safe routes [39].

### Strengths and limitations

The study findings are transferable and generalizable to urban ED settings in western countries. A strength of this study is the prospective approach to systematically screen for alcohol among e-scooter riders presenting to the ED. To the best of our knowledge, the proportion of missing alcohol data is lower than in previous studies, allowing an analysis of alcohol involvement and injury pattern. A limitation is that breathalyser test results may turn negative if there is a long delay from the incident to the ED arrival. However, most of the participants arrived shortly after the incident due to the study hospital´s central city location. One may argue that serum ethanol is a more accurate test, since breathalysers depend on patient collaboration. However, blood samples are rarely required for ED treatment of e-scooter injuries, therefore we considered the non-invasive breathalyser to be a more ethical and practical choice.

Another limitation is that participants may have declined to take the alcohol test due to the risk of being denied accident insurance compensation, which may also have caused some ED staff members to refrain from asking the patient to take the test. This may be avoided in future studies by entering the test results in a protocol separate from the patients’ medical records. Furthermore, the City of Stockholm announced the intervention on rental e-scooters less than two months before its introduction. By that time this study had been approved by the Swedish Ethical Review Authority with a calculated sample size and study period that were based on the number of e-scooter injuries in 2021. Consequently, the 2022 sample turned out smaller than was predicted and did not have enough power to detect differences in mean BAC depending on the arrival time and day among the test positive participants.

## Conclusion

This study found that six out of ten e-scooter riders arriving to the ED nighttime had used alcohol and that eight out of ten sustained face and head injuries when alcohol was involved. We also found a decrease of e-scooter injuries in the study ED and the total number of rental e-scooter trips in the city by one third after the introduction of a city limit on the maximum number of rental e-scooters. Future research should evaluate interventions to prevent intoxicated e-scooter riding nighttime and enhance helmet use.

## Data Availability

The datasets generated and analysed during the current study are available from the corresponding author on reasonable request.

## Appendix 1. Missing data on alcohol involvement - analysis of participant characteristics

**Table:**

	All presentations (*n* = 133)			Within 24 h (*n* = 126)	
Alcohol involvement	Unknown	Known		Not tested	Tested
**N (%)**	25 (18.8)	108 (81.2)		37 (29.4)	89 (70.1)
**Age**					
Mean (SD), years	35.4 (10.2)	33.9 (13.8)		32.2 (11.7)	34.7 (13.9)
95% CI, years	31.2–39.6	31.2–36.5		28.3–36.1	37.7–37.6
		*p* = 0.236			*p* = 0.347
**Sex**					
Female, n (%)	11 (44)	34 (31)		17 (46)	28 (31)
Male, n (%)	14 (56)	74 (69)		20 (54)	61 (69)
95% CI, male %	35–77	60–77		37–71	59–78
		*p* = 0.233			*p* = 0.122
**Arrival time**					
06:00–21:59, n (%)	15 (60)	50 (46)		19 (51)	40 (45)
22:00–05:59, n (%)	10 (40)	58 (54)		18 (49)	49 (55)
95% CI, 22:00–05:59 (%)	19–61	44–63		32–66	45–66
		*p* = 0.217			*p* = 0.512
**Arrival day**					
Monday to Friday, n (%)	19 (76)	74 (69)		27 (73)	60 (67)
Saturday to Sunday, n (%)	6 (24)	34 (31)		10 (27)	29 (33)
95% CI, Saturday to Sunday (%)	6–42	23–40		12–42	23–43
		*p* = 0.462			*p* = 0.539

## Appendix 2. Abbreviated injury scale (AIS) and injury severity score with and without alcohol involvement

**Table:**

Maximum AIS per participant	Alcohol involvement			
	No	Yes	Missing	Sum
1	22	39	11	**72**
2	24	14	14	**52**
3	5	4	0	**9**
≥ 4	0	0	0	**0**
**Sum**	**51**	**57**	**25**	**133**
**Injury Severity Score**	**Alcohol involvement**			
	**No**	**Yes**	**Missing**	**Sum**
1	14	24	5	**43**
2	7	13	4	**24**
3	1	2	2	**5**
4	12	9	9	**30**
5	9	3	5	**17**
6	1	1	0	**2**
7	0	0	0	**0**
8	2	0	0	**2**
9	4	2	0	**6**
10	0	1	0	**1**
11	0	0	0	**0**
12	0	1	0	**1**
13	1	1	0	**2**
≥ 14	0	0	0	**0**
**Sum**	**51**	**57**	**25**	**133**

## Abbreviations

AIS: Abbreviated Injury Scale
BAC: Blood Alcohol Concentration
CI: Confidence Interval
ED: Emergency Department
E-scooter: Electric scooter
ISS: Injury Severity Score
STRADA: Swedish Traffic Accidents Data Acquisition
